# Preventative Effects of Caffeic Acid Phenyl Ester on Cadmium Intoxication Induced Hematological and Blood Coagulation Disturbances and Hepatorenal Damage in Rats

**DOI:** 10.1155/2014/764754

**Published:** 2014-03-30

**Authors:** Tariq Helal Ashour

**Affiliations:** Department of Laboratory Medicine, Faculty of Applied Medical Sciences, Umm Al-Qura University, P.O. Box 7607, 7152 Makkah, Saudi Arabia

## Abstract

The preventative effect of caffeic acid phenyl ester (CAPE) against hematological, blood coagulation, and hepatorenal disturbances in cadmium (Cd) intoxication was investigated in rats. Male Wistar rats were randomly assigned into control group, Cd-group, and Cd + CAPE group. Cd intoxication was induced by intraperitoneal injection (i.p.) of CdCl_2_ (1 mg/kg/day) for 21 days, and CAPE was daily given (10 micromol/kg; i.p.) for also 21 days. The results showed that Cd intoxication impaired hepatorenal function and significantly prolonged prothrombin time and activated partial thromboplastin time and decreased fibrinogen level, red blood cells and platelets counts, hemoglobin concentration, hematocrit, mean corpuscular volume, mean corpuscular hemoglobin, and mean corpuscular hemoglobin concentration. Interestingly, all these hematological, blood coagulation, and hepatorenal deteriorations of Cd toxicity were significantly prevented by CAPE. Additionally, CAPE significantly reversed the significant decreases in levels of total reduced glutathione and superoxide dismutase and increases in levels of thiobarbituric acid reactive substances that were observed in the sera and liver and kidney homogenates of Cd group. It is concluded that CAPE is a promising compound that can counteract the hematological and blood coagulation disturbances, oxidative stress, and hepatorenal damages in Cd intoxication. However, further studies are crucially needed to improve this treatment in patients.

## 1. Introduction

Caffeic acid phenyl ester (CAPE) is one of the main medicinal components of honeybee propolis that possesses a variety of biological and pharmacological actions such as potent free radical scavenging, antioxidant, anti-inflammatory, cytoprotective, immunomodulatory, and antiviral and promising anticancer properties [1]. Also, the ameliorating and counteracting effects of CAPE on different disease modalities of hematological, blood coagulation, and vascular abnormalities have recently been emerged. In this concept, CAPE had shown to ameliorate blood coagulation abnormalities and disturbed oxidative stress in endotoxic model of acute liver failure [2], increase cerebral blood flow and improve ischemic stroke in neurovascular disease [3], protect peripheral blood mononuclear cells against hyperthermal stress [4], prevent drugs to induce toxic and damage effects on red blood cells [5], and potently inhibit the synthesis of inflammatory and atherosclerotic leukotrienes in human polymorphonuclear leukocytes and whole blood [6].

Cadmium (Cd) is classified as a very harmful environmental pollutant to the humans that transfers between various levels of the food chain [7]. Though the definite mechanisms of its associated toxicity are not yet well covered, it has been revealed that Cd markedly stimulates the formation of reactive oxygen species (ROS), enhances lipid peroxidation and cell membrane damage, and depletes the antioxidant defense elements in different body organs [8]. It has been proved that after exposure, Cd enters the blood and binds to the erythrocyte membranes and blood albumin and then is transported to liver, where it bounds to metallothionein (MT) [9]. The Cd-MT complex is then released back into circulation [9] and accumulates in the blood system, kidney, liver, lung, testis, brain, and bone [10]. In the blood and tissues, Cd stimulates the formation of ROS, thus causing oxidative damage, which results in a loss of cell membrane functions [11], multiorgan damage, and important hematological alterations [12, 13].

Over the past decade, a variety of research studies have reported that medications with free-radical scavengers and antioxidants are useful in protecting against Cd toxicity [7, 13, 14]. To date, few studies have shown the remarkable tissue protective effects of CAPE against cadmium intoxication. In this regard, therapy with CAPE had significantly resulted not only in elimination of Cd from blood and tissues but also in preventing Cd-induced oxidative stress, overproduction of ROS, impaired cellular ultrastructures, and injuries in the renal, cardiac, and liver tissues [8, 15–17]. However, the possible preventative effect of CAPE against the hematological and blood coagulation dysfunctions secondary to Cd intoxication is still not well investigated. Coherently, the present study aimed to investigate the possible alleviating effects of CAPE on the altered hematological and coagulopathy state as well as the oxidative stress response that could be associated with Cd intoxication in rats.

## 2. Materials and Methods

### 2.1. Chemicals and Reagents

Cadmium chloride (CdCl_2_) and caffeic acid phenethyl ester (CAPE) were purchased from Sigma-Aldrich Chemical Company (St. Louis, Missouri, USA). Commercial assay kits of total reduced glutathione (GSH) content, superoxide dismutase (SOD) activity, and thiobarbituric acid reactive substances (TBARS) concentration were purchased from Cayman Chemical (Ann Arbor, Michigan, USA). All other used chemicals and reagents were of analytical grade and obtained from standard commercial supplies as stated under the sections of their applications.

### 2.2. Animals, Treatments, and Experimental Approach

Forty adult male Wistar albino rats, weighing 230–250 g, were used in the present study. The rats were housed five per cage under controlled temperature (20–25°C) and 12 h light-dark cycle and allowed free access to water and a commercial rat pellets stock diet. All experimental protocols were approved by the Committee for the Care and Use of Laboratory Animals at Umm Al-Qura University, KSA, and all animals received care according to the criteria outlined in the Guide for the Care and Use of Laboratory Animals prepared by the National Academy of Sciences and published by the National Institutes of Health. The rats were randomly divided into 3 experimental groups: control group (*n* = 10), Cd-group (*n* = 15), and Cd plus CAPE group (*n* = 15). In Cd and Cd + CAPE groups, CdCl_2_, dissolved in physiological saline (0.9% sodium chloride in distilled water), was intraperitoneally injected at a dose of 1 mg/kg/day for 21 days, and in Cd + CAPE group, CAPE was coadministered i.p. at a dose of 10 micromol/kg for also 21 days. The doses of both Cd and CAPE were chosen on the basic of previous studies [2, 8, 15]. Control rats were received only with physiological saline. At the end of the study (i.e., at day 22), all animal groups were fasted for 12 h and then sacrificed under ether anesthesia and their blood specimens were collected. After blood withdrawal, the livers and kidneys were harvested quickly, and a portion of each isolated organ was homogenized in RIPA lysis buffer (1 : 6 w : v) and then centrifuged at 10,000 rpm for 10 min at 4°C. The obtained supernatant was used for measuring the intrahepatic and intrarenal concentrations of oxidative stress and antioxidant biomarkers as described below.

### 2.3. Hematological and Blood Coagulation Analysis

During scarification process, three blood samples were immediately withdrawn from the vena cava of each rat and used for blood coagulation, hematology, and biochemical analyses. The first sample was collected on a tube contained 0.11 M sodium citrate anticoagulant (1 : 9, v : v) and used for plasma preparation for screening of the following blood coagulation tests: prothrombin time (PT), activated partial thromboplastin time (APTT), and fibrinogen concentrations (FIB), by using Dade Behring reagents and following manufacturer's instructions as previously described [18]. 

The second sample was collected in a tube contained disodium salt of ethylene diamine tetra acetic acid (EDTA) anticoagulant and used for determination of the following hematology parameters: counts of red blood cells (RBCs), white blood cells (WBCs) and platelets (PLTs), hemoglobin (HGB) concentration, hematocrit (HCT), mean corpuscular volume (MCV), mean corpuscular hemoglobin (MCH), and mean corpuscular hemoglobin concentration (MCHC). These hematological parameters were determined by standard hematological techniques. The last portion of the collected blood was placed in a plain centrifuge tube without any anticoagulant and after centrifugation process, its corresponding serum was obtained and used for the below mentioned assays.

### 2.4. Biochemical Analysis

The prepared sera samples were employed for measurement of the serum concentrations of liver function enzymes (aspartate aminotransferase (AST), alanine aminotransferase (ALT) and alkaline phosphatase (ALP)), albumin (ALB), and kidney function biomarkers (serum creatinine (CRE), and blood urea nitrogen (BUN)).

### 2.5. Evaluation of Antioxidant and Oxidative Stress Status

The levels of total reduced glutathione (GSH) and activities of superoxide dismutase (SOD) (as indices of nonenzymatic and enzymatic antioxidant status, resp.) were measured in the serum and homogenates of the liver and kidney tissues. On the other hand, the concentrations of thiobarbituric acid reactive substances (TBARS), indices of lipid peroxidation, and oxidative stress were also determined in these sera, liver, and renal biological samples. During these three assays, specific commercial kits (Cayman Chemical; Ann Arbor, Michigan, USA) were used, and all samples were processed in duplicate and according to the manufacturer's instructions.

### 2.6. Statistical Analysis

The results were expressed as the mean ± standard deviation (SD) and statistical analysis was carried out using SPSS software, version 16.0 (SPSS Inc., Chicago, IL, USA). One-way analysis of variance (ANOVA) followed by a Student's* t*-test were used to analyze the statistical differences. *P* < 0.05 was considered to represent a statistically significant difference.

## 3. Results

### 3.1. Hematological and Blood Coagulation Findings

The hematological and blood coagulation changes are shown in Table 1. Compared with the control group, Cd-group had showed significant decreases in RBC and PLT counts, but not in WBC counts. Moreover, the values of HGB, HCT, MCV, MCH, and MCHC had significantly decreased in Cd-group in comparison with their values in the normal controls. In contrary, therapy with CAPE had successfully ameliorated these hematological toxic effects of Cd, whereby the counts of RBCs and PLTs as well as the values of HGB, HCT, MCV, MCH, and MCHC had showed no significant differences than those of normal control rats.

Intoxication with Cd had also resulted in significant deteriorations on blood coagulation. As demonstrated in Table 1, there was significant prolongation of PT and APTT clotting tests associated with significantly decreased fibrinogen levels in Cd-group, when compared with control group. By contrast, treatment of these Cd-injected rats with CAPE had significantly succeeded in improving the values of PT APTT and fibrinogen (Table 1). Collectively, this part of the results indicates that Cd intoxication in rats is associated with important alterations in the hematological and blood coagulation parameters, and these alterations can remarkably be alleviated by CAPE therapy.

### 3.2. Biochemical Findings

The results of serum biochemistry reflect that both the hepatic and renal function of rats had been altered due to Cd intoxication and these damaging effects of Cd had been attenuated by CAPE therapy. As shown in Table 2, compared with the control group, significant changes in the serum levels of AST, ALT, ALP, and ALB (as biomarkers of liver function), as well as the serum levels of CRE and BUN (as biomarkers of renal function), were detected only in Cd group but not in Cd + CAPE group.

### 3.3. Serum, Renal, and Hepatic Levels of GSH, SOD, and TBARS

Level of GSH (an example of nonenzymatic antioxidant defense mechanism), activity of SOD (an example of enzymatic antioxidant defense mechanism), and concentration of TBARS (an index of lipid perioxidation and oxidative stress) were measured in the sera samples and liver and kidney tissue homogenates of all animal groups. As demonstrated in Table 3, Cd intoxication in rats had associated with significant reduction in GSH content and SOD activity, as well as marked elevation in TBARS content in the sera, livers, and kidneys of Cd group. On the other hand, concurrent administration of CAPE with Cd had obviously counteracted these effects of Cd on GSH, SOD, and TBARS in all tested biological samples.

## 4. Discussion

Cadmium (Cd) is classified as a highly toxic environmental pollutant to the humans and animals. Caffeic acid phenyl ester (CAPE) is a biological active component of honeybee propolis extracts with potent free radical scavenger and antioxidant activity [8]. This study was designed to investigate the effect of CAPE on some hematological parameters, blood coagulation, and hepatorenal functional status in cadmium (Cd) intoxication in rats. The results showed the remarkable protective effect of CAPE against Cd-induced hepatorenal injury with marked hematological and blood hemostasis disturbances in rats.

There is a compelling body of evidence that Cd exposure importantly contributes to human and animal liver and kidney diseases [19, 20]. After the intake and resorption, Cd enters the blood and binds to the erythrocyte membranes and plasma albumin [21]. In the blood and tissues, Cd stimulates lipid peroxidation and the formation of ROS, thus causing oxidative cellular and tissue damages [9, 11, 12]. In support, the biochemical findings of the present study revealed the occurrence of hepatorenal impairment in Cd-exposed animals but not in Cd + CAPE-treated animals, as reflected by significant elevations in the serum levels of AST, ALT, ALP, creatinine, and BUN [22]. The observed hepatorenal protective effects of CAPE against Cd were also accompanied with preventing Cd to induce lipid peroxidation and decrease antioxidant defense system. Taken together, these findings can reinforce those previously reported that CAPE has a potent protective effect against renal, liver, and other body organ injuries caused by Cd intoxication, and this might be by inhibiting Cd-induced oxidative stress and tissue damage [8, 15–17].

Hematopoietic system is one of the most sensitive systems to evaluate the hazards effects of poisons and drugs in humans and animals [22]. In consistency, the current study indicated that an exposure to toxic Cd was associated with significant hematological disturbances and therapy with CAPE had successfully alleviated these hematological changes induced by Cd (Tables 1–3). In this regard, anemia was clearly observed in rats received Cd alone but not in those received Cd plus CAPE. In agreement, it has been approved that Cd accumulation in kidney, liver, and spleen can suppress the activity of these important hematopoietic tissues [23]. Additionally, some previous studies revealed that exposure to Cd induces anemia associated with decrease in RBCs counts and induction of oxidative damage and lipid peroxidation in blood and RBCs [12, 13, 24]. Moreover, the decreases that were observed her in the values of MCV, MCH, and MCHC in Cd group but not in Cd + CAPE group can also indicate the further ability of Cd intoxication to induce microcytic hypochromic anemia [20], and this effect was also alleviated by CAPE therapy. Collectively, Cd intoxication might lead to anemia as a result of either suppression the activity of hematopoietic tissues, impaired erythropoiesis, and accelerated erythroclasia because of the altered RBCs membrane permeability, increased RBCs mechanical fragility, and/or defective Fe metabolism [20].

Data of blood clotting tests demonstrated that exposure to Cd had resulted in a significant hypocoagulation state in form of marked prolongation of the coagulation tests PT, APTT, low platelet count, and decreased fibrinogen levels and this Cd's hemostatic dysregulation effect was significantly improved by CAPE treatment. It is well known that the liver is the major organ for synthesis of procoagulation factors and substances. Thus, the hypocoagulation state that was observed her in Cd group but not in Cd + CAPE group might be related to Cd-induced liver injury with decreased production of the procoagulation factors and reduced hepatic clearance of plasminogen activators leaded to enhanced fibrinolytic activity [2, 25]. These findings and its related suggestion can also be supported by Korish's report [2] that therapy with CAFÉ protects the liver and prevents the hemostatic alterations in endotoxic-induced acute liver failure.

Earlier studies had indicated that treatment with free-radical scavengers and antioxidants are useful in protecting against Cd toxicity [7, 13, 14]. Therefore, depletion of antioxidative defense mechanism (represented by decreased SOD and GSH) together with increased TBARs (an index of lipid peroxidation and oxidative stress) that were observed in the serum, liver, and kidney tissues of Cd-injected rats but not of Cd-injected/CAPE-treated rats could be the main underlying pathogenic mechanisms by which the injected Cd had induced its hematological and organ toxicity [8, 13]. Similarly, the preventative effects of CAPE that were also observed here against Cd could also be attributed to its potent antioxidant property [2, 26]. In harmony, Gokalp et al. [5] showed the cytoprotective ability of CAPE in preventing anti-TB drug “isoniazid” to induce oxidative damage in RBCs, and the potent renoprotective effect of CAPE against Cd-induced injury has been previously confirmed and attributed mainly to the antioxidant activity of CAPE [8, 15–17]. Finally, by increasing the antioxidant elements and inhibiting the oxidative status, CAPE had shown to protect the brain vasculature from ischemic stroke disease [3] and protect the liver and improve blood coagulation abnormalities in endotoxic model of acute liver failure [2].

## 5. Conclusion

Based on the presented results, it can be concluded that Cd intoxication was resulted in development of hematological and hemostatic alterations, as well as hepatorenal dysfunctions in rats, and this might be due to enhancing of lipid peroxide concentration and/or depletion of the activity of antioxidant defense elements. Furthermore, the ability of CAPE to protect against the hematological, blood coagulation, and hepatic and renal toxic effects of Cd could also be attributed to restore the antioxidant activity and/or inhibit lipid peroxidation in both blood and organs. Therefore, CAPE could be a promising agent for the treatment of Cd intoxication; however, further studies are crucially needed to improve this treatment in patients.

## Figures and Tables

**Table 1:** Hematological and blood coagulation findings.

Parameter	Units	Control group (*n* = 10)	Cd group (*n* = 15)	Cd + CAPE group (*n* = 15)
WBC	10^3^/*μ*L	11.92 ± 0.79	13.53 ± 2.72	12.27 ± 1.46
RBC	10^6^/*μ*L	8.67 ± 0.48	5.93 ± 0.83*	8.10 ± 0.16^#^
HGB	g/L	157.67 ± 7.23	115.46 ± 6.03∗	151.67 ± 3.51^#^
HCT	%	48.33 ± 0.71	41.83 ± 1.00*	46.13 ± 1.01^#^
MCV	fL	64.27 ± 0.84	52.87 ± 4.48*	60.20 ± 1.54^#^
MCH	pg	22.13 ± 1.65	16.70 ± 1.11*	20.40 ± 1.20^#^
MCHC	g/L	344.42 ± 39.50	300.26 ± 27.77*	337.67 ± 38.50^#^
PLT	10^3^/*μ*L	1000.27 ± 91.83	775.32 ± 59.61*	933.36 ± 70.76^#^
PT	Second	12.82 ± 1.37	43.14 ± 4.33*	17.76 ± 2.33^#^
APTT	Second	21.73 ± 3.46	77.52 ± 13.43*	29.36 ± 5.21^#^
FIB	mg/dL	263.90 ± 33.77	93.37 ± 9.37*	226.68 ± 42.23^#^

The values are presented as means ± SD. (Cd) cadmium, (CAPE) caffeic acid phenyl ester, (WBC) white blood cell, (RBC) red blood cell, (HGB) hemoglobin, (HCT) hematocrit, (MCV) mean corpuscular volume, (MCH) mean corpuscular hemoglobin, (MCHC) mean corpuscular hemoglobin concentration, (PLT) platelet, (PT) prothrombin time, (APTT) activated partial thromboplastin time, and (FIB) fibrinogen. *P < 0.05 versus control group; ^#^ *P* < 0.05 versus Cd group.

**Table 2:** Serum levels of liver and kidney function biomarkers.

Parameter	Units	Control group (*n* = 10)	Cd group (*n* = 15)	Cd + CAPE group (*n* = 15)
AST	IU/L	109.00 ± 12.17	571.33 ± 72.29*	163.00 ± 14.93^#^
ALT	IU/L	49.17 ± 3.27	136.07 ± 21.06*	73.33 ± 11.35^#^
ALP	IU/L	206.33 ± 20.79	274.33 ± 25.14*	211.33 ± 17.35^#^
ALB	g/dL	4.33 ± 0.59	2.86 ± 0.38*	4.01 ± 0.27^#^
CRE	mg/dL	0.26 ± 0.04	0.41 ± 0.34*	0.29 ± 0.01^#^
BUN	mg/dL	47.36 ± 9.12	63.65 ± 13.44*	49.22 ± 10.10^#^

The values are presented as means ± SD. (Cd) cadmium, (CAPE) caffeic acid phenyl ester, (AST) aspartate aminotransferase, (ALT) alanine aminotransferase, (ALP) alkaline phosphatase (ALP), (ALB) albumin, (CRE) creatinine, and (BUN) blood urea nitrogen. *P < 0.05 versus control group; ^#^ *P* < 0.05 versus Cd group.

**Table 3:** Antioxidant and lipid peroxidation status in serum and hepatorenal tissues.

Group	GSH (*μ*mol/mg protein)			SOD (U/mg protein)			TBARS (nmol/mg protein)		
	Serum	Liver	Kidney	Serum	Liver	Kidney	Serum	Liver	Kidney
Control (*n* = 10)	13.7 ± 3.1	69.9 ± 7.3	57.7 ± 9.2	3.8 ± 0.6	289.5 ± 37.3	267.7 ± 23.4	27.4 ± 4.2	48.6 ± 6.2	37.4 ± 5.9
Cd (*n* = 15)	4.7 ± 0.9*	11.7 ± 1.2*	13.74 ± 1.7*	1.2 ± 0.2*	64.8 ± 8.2*	71.5 ± 6.7*	116.2 ± 23.4*	1019.5 ± 195.9*	1113.3 ± 195.9*
Cd + CAPE (*n* = 15)	12.2 ± 1.2^#^	75.9 ± 11.4^#^	68.7 ± 10.2^#^	3.3 ± 0.8^#^	305.3 ± 50.5^#^	311.5 ± 47.8^#^	36.74 ± 5.8^#^	87.2 ± 14.6^#^	118.6 ± 23.29^#^

The values are presented as means ± SD. (Cd) cadmium, (CAPE) caffeic acid phenyl ester, (GSH) total glutathione, (SOD) superoxide dismutase, and (TBARS) thiobarbituric acid reactive substances. *P < 0.05 versus control group; ^#^ *P* < 0.05 versus Cd group.
